# Case Report: Diffuse pulmonary lymphangiomatosis in a child

**DOI:** 10.3389/fped.2025.1591611

**Published:** 2026-01-05

**Authors:** Ya-nan Zhang, Zhen Yuan, Jin-chen Du, Bin Liu, Fu Li, Shou-Cai Hu, Qing-Xin Li

**Affiliations:** 1 Department of Thoracic Surgery, The 940th Hospital of Joint Logistics Support Force of Chinese People’s Liberation Army, Lanzhou, Gansu, China; 2 The First Clinical Department of Gansu University of Traditional Chinese Medicine, Lanzhou, Gansu, China

**Keywords:** diffuse pulmonary lymphangiomatosis (DPL), generalized lymphatic anomaly (GLA), pediatric case, diagnostic imaging, histopathology, mTOR inhibitors

## Abstract

We report a rare case of generalized lymphatic anomaly (GLA) in a 12-year-old male, presenting with intermittent cough, expectoration, and hemoptysis exacerbated by strenuous activity. Key features included bilateral pleural/pericardial effusions; chest/abdominal CT showed diffuse mediastinal infiltration, bronchovascular/interlobular septal thickening, and extrathoracic extension to periaortic abdominal tissues. Definitive diagnosis was confirmed by thoracoscopic biopsy and immunohistochemistry (CD31+, D2-40+, Ki-67 ≈ 3%), differentiating it from mimics like pulmonary lymphangiectasia. The patient achieved symptomatic remission after pericardiectomy, thoracic catheter drainage, and postoperative sirolimus. This case enriches pediatric GLA literature, highlights multidisciplinary diagnosis value, and supports mTOR inhibitors’ role in pediatric GLA management.

## 1 Introduction

Diffuse pulmonary lymphangiomatosis (DPL) is a rare, proliferative disorder of the lymphatic system characterized by multifocal involvement of intrathoracic structures, including the lungs, mediastinum, and pleura (1, 2). Its nonspecific clinical presentation and variable imaging features frequently result in diagnostic delays or misdiagnosis (3), particularly in pediatric populations. Histopathological confirmation remains the gold standard for diagnosis (4); However, as our understanding of lymphangiomatosis has deepened, it has become evident that the disease often exhibits a diffuse, multifocal distribution beyond the thorax. The International Society for the Study of Vascular Anomalies (ISSVA), in its 2018 classification, The term “generalised lymphatic abnormality (GLA)” is suggested to summarise this multisystemic presentation. Consequently, what was previously termed DPL is now more accurately understood as the thoracic manifestation of GLA. This nosological shift from DPL to GLA has profound implications for clinical practice. It urges clinicians to move beyond a localized thoracic diagnosis and actively screen for potential subclinical lesions in other organ systems. Furthermore, it aligns with the evolving treatment paradigm, which increasingly favors systemic pharmacological therapies (e.g., mTOR inhibitors like sirolimus) over localized surgical interventions, targeting the underlying pathogenic process throughout the body. Despite its benign histological appearance, GLA often demonstrates aggressive clinical behavior (5), leading to debilitating complications such as massive pleural and pericardial effusions. Current literature remains sparse, with only limited case reports describing its heterogeneous manifestations and the empirical use of pharmacologic or surgical interventions. By integrating clinical, radiological, and pathological insights, this study seeks to raise awareness of GLA and contribute to the evolving understanding of its diagnosis and management.

## 2 Case description

A 12-year-old boy was admitted with a one-month history of intermittent cough and sputum production, which had worsened over the preceding week and was accompanied by hemoptysis. He reported paroxysmal coughing and sputum production without obvious triggers. At a local hospital, he had initially been prescribed anti-influenza medication, which provided temporary symptom relief. However, after discontinuation, his symptoms exacerbated during strenuous physical activity, presenting with white, foamy sputum, occasional hemoptysis, retrosternal pain, and fever. Chest CT revealed bilateral interstitial pulmonary edema, along with bilateral pleural and pericardial effusions. Initial laboratory results were as follows: white blood cells (WBC): 9.67 × 10^9^ /L, hemoglobin (Hb): 152 g/L, neutrophil percentage: 0.76%, lymphocyte percentage: 0.16%, eosinophil percentage (EOS): 1.7%, platelets (PLT): 256 × 10^9^ /L, and C-reactive protein (CRP): 5.38 mg/L. Due to progressive symptoms, the patient was transferred to our hospital for further evaluation and treatment. On admission, his vital signs were: heart rate: 94 beats/min, respiratory rate: 22 breaths/min, and blood pressure: 92/65 mmHg. Physical examination revealed coarse breath sounds with scattered wet rales bilaterally. Cardiac examination showed a regular rhythm, distant and muffled heart sounds, and no pathological murmurs over the valvular regions.

## 3 Diagnostic assessment

A pericardial drainage procedure yielded 360 mL of non-coagulable pericardial fluid for analysis. The PPD test was negative. Laboratory results showed markedly elevated D-dimer (12.46 mg/L) and fibrin degradation product levels (144.40 μg/ml), with an erythrocyte sedimentation rate of 3.0 mm/h. Biochemical analysis of the pericardial effusion revealed: total protein 71.9 g/L, chloride 108.6 mmol/L, glucose 5.36 mmol/L, and adenosine deaminase 11.6 U/L. Cytological examination identified numerous erythrocytes, scattered mesothelial cells, and lymphocytes.

Repeat chest and abdominal CT scans were performed for detailed evaluation. The thoracic imaging revealed characteristic features suggestive of a systemic lymphatic disorder:

1. Mediastinal and Hilar Infiltration: There was an irregular, homogeneous soft tissue mass infiltrating the mediastinum, which encased major mediastinal vessels and exhibited poor demarcation from adjacent tissues. This finding is indicative of diffuse lymphatic proliferation.
2. Lung Parenchyma Changes: The initially described “interstitial pulmonary edema” was more specifically characterized by diffuse, smooth thickening of the bronchovascular bundles and interlobular septa. Additionally, patchy ground-glass opacities were observed bilaterally. These changes reflect the proliferation of lymphatic channels within the pulmonary interstitium and lymphatic obstruction.
3. Serous Effusions and Membrane Involvement: Bilateral pleural effusions and a pericardial effusion were present, accompanied by visible thickening of the pleural and pericardial membranes. This is a classic manifestation of GLA, resulting from the involvement of serosal surfaces by abnormal lymphatics and impaired drainage of lymphatic fluid.
4. Extrathoracic Extension: Abdominal imaging revealed blurred fat planes and multiple nodular soft tissue lesions surrounding the abdominal aorta and its branches, confirming the systemic nature of the disease.

**Figure 1:**
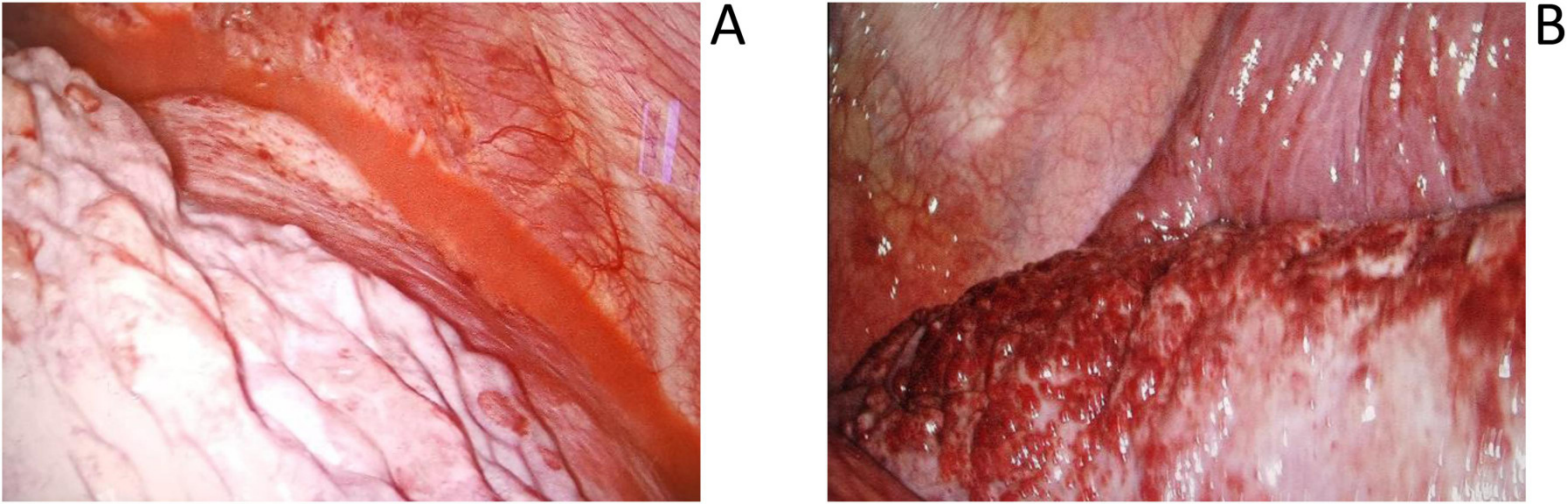
**(A)** Demonstrates substantial pink pleural effusion within the patient's thoracic cavity during surgery, whilst **(B)** reveals red granular nodules on the pulmonary surface.

Collectively, these imaging findings—particularly the combination of a diffusely infiltrating mediastinal mass, smooth interlobular septal thickening, chylous effusions with membrane thickening, and evidence of extra-thoracic involvement—provided critical clues and strongly pointed towards the diagnosis of GLA. This radiological suspicion guided the subsequent decision to pursue tissue confirmation. The patient was subsequently referred to our department for further evaluation and underwent thoracoscopic mediastinal mass excision biopsy, lung wedge resection biopsy, pericardial biopsy, and pericardiectomy. Intraoperatively, a large volume of serous fluid was present in the right thoracic cavity. Numerous red granular nodules were observed on the parietal pleura. The anterior mediastinal mucosa appeared thickened, edematous, and indurated, with multiple red granular nodules on its surface. A substantial amount of reddish effusion was also noted within the pericardium (Figure 1). Frozen section analysis revealed mediastinal hyperplasia with edema, increased vascular density within fibrous tissue, and numerous hemosiderin-laden macrophages in the alveolar cavities of the right lower lobe. Localized alveolar septal fibrosis was also present. Pericardial tissue exhibited fibrous hyperplasia with infiltration of lymphomononuclear cells, neutrophils, and proliferative dilated blood vessels.

**Figure 2:**
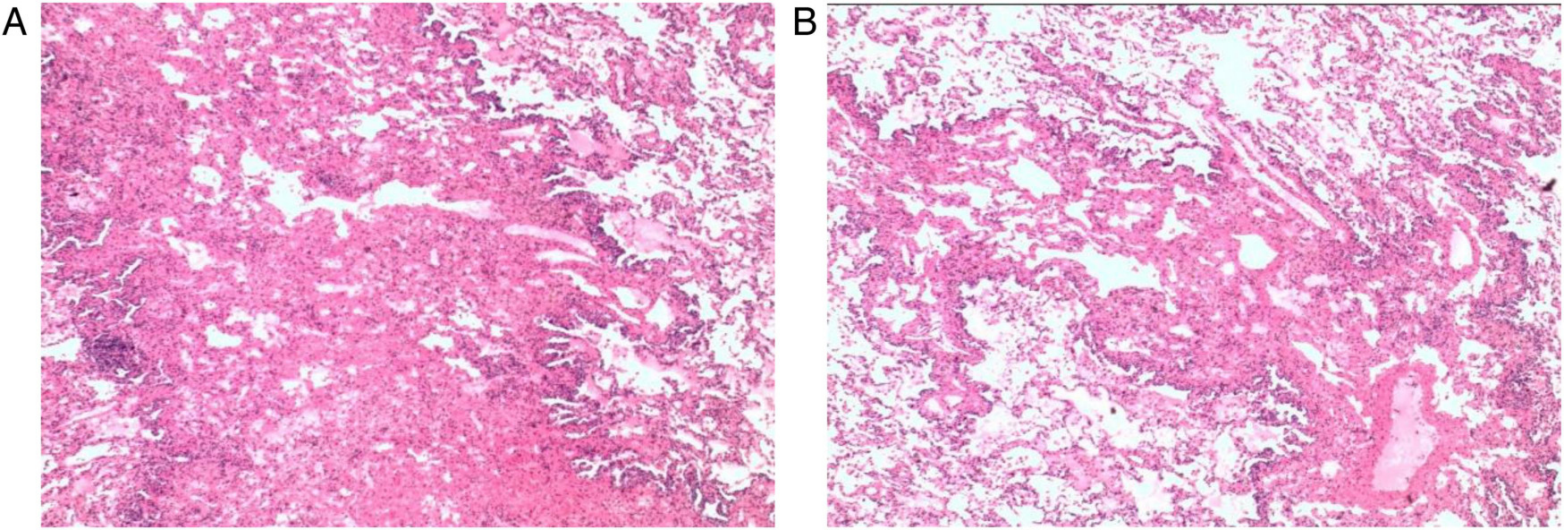
**(A,B)** Presents post-operative histopathological staining of microscopically distorted lymphatic ducts.

**Figure 3:**
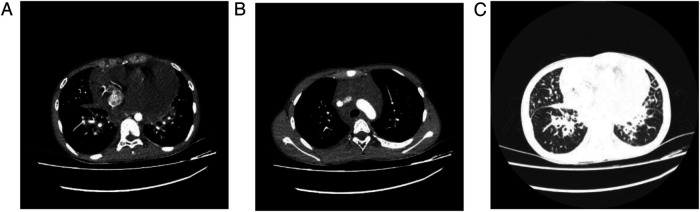
**(A)** Demonstrates extensive pleural effusion and pericardial effusion on the patient's pulmonary CT scan; **(B)** reveals interstitial thickening; and **(C)** shows diffuse ground-glass opacities within the lungs.

Postoperative histological examination confirmed lymphangiomatosis involving the mediastinum, right lower lobe of the lung, and pericardium, characterized by numerous tortuous and dilated lymphatic vessels (Figure 2). Immunohistochemical analysis demonstrated the following profile: CD31 (+), D2-40 (+), TIF-1 (+), CKp (−), CD34 (+), SMA (+), Ki-67 ≈ 3% (Figure 3), NTRK (−), and S100 (−). Integrating the clinical presentation, imaging findings, and pathological results, a final diagnosis of GLA was established.

## 4 Discussion

GLA is a rare, insidious disorder (unknown pathogenesis) that primarily affects children—earlier onset correlates with poorer prognosis (6). This case is unique in that strenuous activity exacerbated symptoms (a rarely reported trigger), and systemic imaging (abdominal CT) confirmed extrapulmonary involvement—key clues for distinguishing GLA from localized lymphatic disorders.

**Figure 4:**
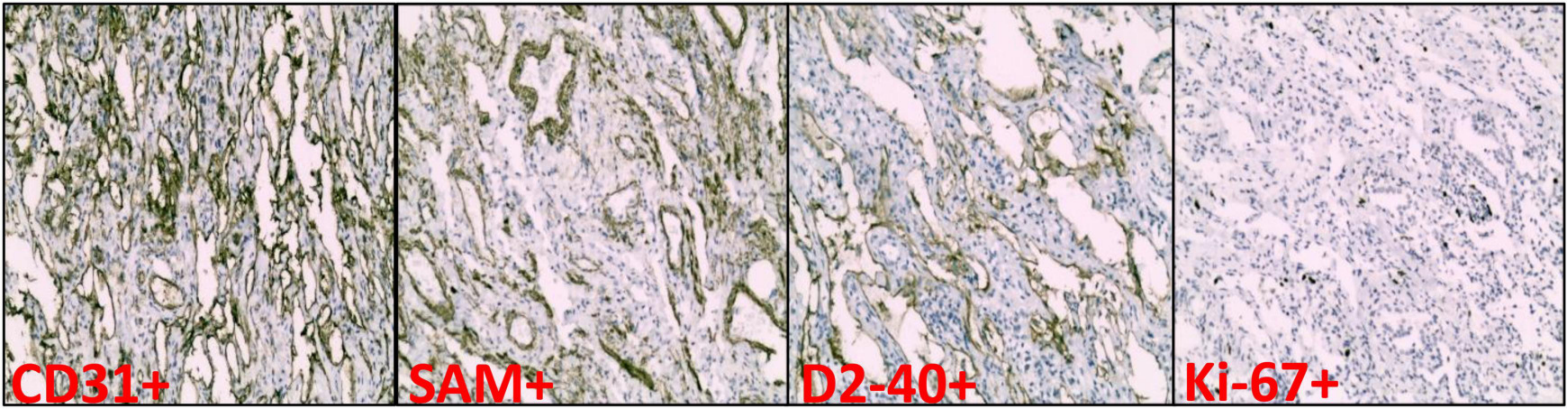
Preoperative chest CT demonstrating interlobular septal thickening, patchy ground-glass shadows, pleural effusion, pericardial effusion, and pericardial thickening.

The imaging characteristics of GLA primarily include soft tissue infiltrative shadows in the mediastinum and hilar regions, thickening of bronchovascular bundles and interlobular septa, patchy ground-glass opacities, pleural effusion with pleural thickening, and pericardial effusion accompanied by pericardial membrane thickening (2). Affected tissues generally appear hypodense, demonstrating water-like attenuation patterns on imaging. Enlarged mediastinal lymph nodes are commonly observed, often accompanied by pleural thickening or effusion. In addition, enlarged hypodense cervical and/or axillary lymph nodes with indistinct margins and minimal enhancement on contrast-enhanced imaging are frequently reported (3). The radiologic findings in the present case are illustrated in Figure 4. Collectively, these imaging features provide important diagnostic clues for GLA.

Although CT imaging of GLA demonstrates relatively specific features, definitive diagnosis requires pathological confirmation, usually through surgical biopsy of mediastinal, pleural, or pulmonary lesions. Intraoperative gross pathology typically reveals abnormal proliferation and dilatation of lymphatic vessels (4). In the present case, the initial diagnostic plan was to perform an endobronchial ultrasound (EBUS)-guided biopsy. However, following multidisciplinary discussion, EBUS was considered to carry a high risk of intraoperative hemorrhage and potential mortality (7). Consequently, thoracoscopic excisional biopsy of the mediastinal mass was performed instead. Postoperative immunohistochemical analysis showed the following profile: CD31 (+), D2-40 (+), TIF-1 (+), CKp (−), CD34 (+), SMA (+), Ki67 ≈3%, NTRK (−), and S100 (−). Notably, endothelial cells in GLA typically express D2-40, CD31, and Factor VIII-related antigens.

GLA must be differentiated from four key mimics:

1. Diffuse pulmonary lymphangiectasia: Primary (congenital connective tissue abnormalities) vs. secondary (post-surgery/radiation); histologically, it shows increased lymphatic vessels but no systemic involvement (unlike GLA's extrapulmonary lesions) (8); 2. Pulmonary capillary hemangiomatosis (PCH): Proliferation of alveolar septal capillaries (not lymphatics), with CT features of centrilobular ground-glass nodules + severe pulmonary hypertension; D2-40 (−) (9); 3. Kaposiform lymphangiomatosis (KLA): Rapid progression, life-threatening coagulopathy, and “Kaposi-like” spindle cell clusters (not seen in this case) (10). The disease commonly involves multiple organs, including the lungs, mediastinum, skin, subcutaneous tissue, trabeculae, and spleen (11, 12). 4. Kaposiform hemangioendothelioma (KHE): Discrete enhancing masses + bone destruction (no such findings in this case), with slit-like vascular spaces (vs. GLA's dilated lymphatics) (13).

Due to the rarity of GLA and the limited number of reported cases globally, there is no standardized treatment protocol. Current management is mostly palliative, focusing on symptom relief (14):

### 4.1 Pharmacological therapy

Propranolol: Downregulates VEGF and other pro-angiogenic factors via beta-adrenergic receptor blockade. Clinical evidence for GLA is scarce, not a mainstream therapy, but may be attempted in selected cases or when other options are unavailable (15, 16). Bevacizumab: Binds and neutralizes VEGF-A to inhibit lymphangiogenesis. Case reports (e.g., Aman et al., Onyeforo et al.) demonstrate favorable outcomes, including improved lung function (FEV1, FVC, DLCO) after 6 months of treatment (17). Imatinib: Targets tyrosine kinases like PDGFR (involved in vascular anomaly pathogenesis). Only a few case reports indicate potential disease stabilization or improvement (18, 19). Sirolimus (mTOR inhibitor) (20, 21): Provides symptomatic relief in most GLA patients, but long-term efficacy requires further evaluation; identifying responsive patients is a key research direction.

### 4.2 Surgical therapy

Surgery is challenging due to the difficulty in distinguishing diseased from normal lymphatic vessels. Complete resection is virtually impossible with high postoperative recurrence rates, so surgery is reserved for managing complications rather than curative purposes.

### 4.3 Case management

The patient, the first GLA case at our hospital, was transferred to a higher-level hospital due to limited local experience. Follow-up via telephone revealed the child underwent thoracic duct release surgery, was discharged with improved chest tightness, and has maintained stable condition while regularly taking sirolimus tablets and linezolid post-discharge.

In summary, both the diagnosis and treatment of GLA remain fraught with challenges. In pediatric patients presenting with unexplained cough, hemoptysis, large-volume chylous pleural effusion, pericardial effusion, and chest CT findings such as diffuse mediastinal or paratracheal soft-tissue infiltration, confluent lymphadenopathy with consolidation, and diffuse thickening of peritracheobronchial bundles and interlobular septa, GLA should be carefully considered. To establish a definitive diagnosis, open or thoracoscopic lung biopsy is recommended whenever feasible, as bronchoscopy carries a substantial risk of hemorrhage (22). Additionally, the timely initiation of symptomatic supportive therapy is critical to slowing disease progression and improving clinical outcomes.

## Data Availability

The raw data supporting the conclusions of this article will be made available by the authors, without undue reservation.
